# Novel Resin-Based Antibacterial Root Surface Coating Material to Combat Dental Caries

**DOI:** 10.3390/jfb15060168

**Published:** 2024-06-19

**Authors:** Nader Almutairi, Abdullah Alhussein, Mohammad Alenizy, Ibrahim Ba-Armah, Jirun Sun, Michael D. Weir, Hockin H. K. Xu

**Affiliations:** 1 PhD Program in Dental Biomedical Sciences, University of Maryland School of Dentistry, Baltimore, MD 21201, USA; nader.almutairi@umaryland.edu (N.A.);; 2 Department of Conservative Dental Sciences, College of Dentistry, Prince Sattam bin Abdulaziz University, Alkharj 16245, Saudi Arabia; 3 Department of Restorative Dental Science, College of Dentistry, King Saud University, Riyadh 11545, Saudi Arabia; aalhussein@ksu.edu.sa; 4 Department of Restorative Dental Sciences, University of Hail, Hail 55475, Saudi Arabia; 5 Department of Restorative Dental Sciences, College of Dentistry, Imam Abdulrahman Bin Faisal University, Dammam 31441, Saudi Arabia; 6 The ADA Forsyth Institute, Cambridge, MA 02142, USA; 7 Department of Biomaterials and Regenerative Dental Medicine, University of Maryland School of Dentistry, Baltimore, MD 21201, USA; 8 Center for Stem Cell Biology & Regenerative Medicine, University of Maryland School of Medicine, Baltimore, MD 21201, USA; 9 Marlene and Stewart Greenebaum Cancer Center, University of Maryland School of Medicine, Baltimore, MD 21201, USA

**Keywords:** coating, resin, antibacterial, oral biofilm, root caries

## Abstract

Root caries caused by cariogenic bacteria are a burden on a large number of individuals worldwide, especially the elderly. Applying a protective coating to exposed root surfaces has the potential to inhibit the development of caries, thus preserving natural teeth. This study aimed to develop a novel antibacterial coating to combat root caries and evaluate its effectiveness using the antibacterial monomer dimethylaminohexadecyl methacrylate (DMAHDM). DMAHDM was synthesized and incorporated into a resin consisting of 55.8% urethane dimethacrylate (UDMA) and 44.2% TEG-DVBE (UV) at a 10% mass fraction of glass filler. Multiple concentrations of DMAHDM were tested for their impact on the resin’s mechanical and physical properties. *S. mutans* biofilms grown on resin disks were analyzed for antibacterial efficacy. Cytotoxicity was assessed against human gingival fibroblasts (HGFs). The results showed an 8-log reduction in colony-forming units (CFUs) against *S. mutans* biofilm (mean ± sd; n = 6) (*p* < 0.05) when 5% DMAHDM was incorporated into the UV resin. There was a 90% reduction in metabolic activity and lactic acid production. A low level of cytotoxicity against HGF was observed without compromising the physical and mechanical properties of the resin. This coating material demonstrated promising physical properties, potent antibacterial effects, and low toxicity, suggesting its potential to protect exposed roots from caries in various dental procedures and among elderly individuals with gingival recession.

## 1. Introduction

Root caries caused by cariogenic bacteria are a burden on a large number of individuals worldwide, especially the elderly. As people are living longer and keeping their natural teeth into old age, the prevalence of root caries is continuously increasing [1,2,3]. A new study has provided an updated assessment of the occurrence of untreated caries in the United States. The findings indicate that 21.3% of the population is affected by untreated caries, with root caries accounting for 10.1% of this incidence [4]. Moreover, the global population of individuals aged 65 and above is estimated to rise from 12% to 22% by the year 2050, with the majority (80%) residing in middle- and low-income nations [5]. Root caries refers to any form of dental caries that develops on the root surface of a tooth [6]. The term refers to a lesion located below the cementoenamel junction (CEJ) that is either non-cavitated or cavitated and does not involve the surrounding enamel. Root caries are characterized by discoloration, softness, and lack of clear demarcation, affecting both the cementum and the underlying dentin, and mainly associated with gingival recession [5]. 

In their natural composition, dentin, and cementum exhibit distinctions from enamel. Dentin and cementum consist of approximately 45% to 50% and 70% inorganic material, respectively, contrasting with the enamel structure [7,8]. The microstructure of hydroxyapatite within dentin and cementum lacks the same level of organization observed in enamel [9]. Moreover, elevated levels of magnesium and carbonate are characteristic of these layers, making them more susceptible to solubility [10]. Consequently, the pH value for mineral dissolution is between approximately 6 and 6.8 in root dentin and cementum, in contrast to the enamel which is pH 5.4 [11,12].

Currently, the primary non-invasive approach for inhibiting root caries involves four agents that have been studied in the literature: fluoride, chlorhexidine, amorphous calcium phosphate (ACP), and silver diamine fluoride. Fluoride is considered the most widely recognized and essential element in dental caries prevention [13]. However, these agents require time and adequate conditions such as optimum fluoride concentration and appropriate pH levels for mineral formation. SDF on the other hand has been used for caries arrest due to the presence of fluoride to remineralize and silver as an antibacterial agent but may discourage patients from continuing the treatment as it causes tooth discoloration [14]. Resin-modified glass ionomers (RMGICs) have been employed in the management of root caries, offering not only remineralizing properties but also the capacity to form chemical bonds with the tooth structure and facilitate fluoride release [5]. Nevertheless, individuals experiencing hyposalivation present a dry oral environment that could potentially undermine the durability of this cement [5]. 

Moreover, resin-based materials have been used and studied in the literature as a treatment option to treat root caries. Resin-based products have limitations, especially challenges in dentin bonding where the hydrophilicity of the resin adhesives makes the material prone to hydrolysis. Triethylene glycol divinylbenzyl ether (TEG-DVBE) is a low-viscosity ether-based monomer that can resist hydrolysis challenging situations. It is used in this formulation as a diluent monomer. The combination of UDMA and TEG-DVBE (UV) (denoted as “UV” in this paper) results in a slow rate of polymerization leading to a delayed gel phase. It is believed that the longer it takes for UDMA/TEG-DVBE to polymerize and reach maximum rigidity, the lower the contraction stress and the greater the relaxation that occurs at the resin interface. The UV resin has a hydrophobic and flexible structure that shows a high degree of conversion. This structure enables the resin to effectively resist enzymatic and hydrolytic degradation, minimize water absorption, and reduce polymerization shrinkage stress [15,16].

The potential incorporation of the antibacterial monomer dimethylaminohexadecyl methacrylate (DMAHDM) into a resin-based composite has been explored in recent studies [16,17]. This monomer possesses the capacity to disrupt the biofilm through a contact-killing mechanism. The positive charge of the quaternary amine group in DMAHDM can interfere with bacterial cell membrane activities, disturb ion exchange, and disrupt the electrical equilibrium of the cell membrane, finally resulting in bacterial mortality. Additionally, the monomer can successfully copolymerize with dental resins, resulting in a material with a long-lasting antibacterial effect [18,19]. No study has reported the use of this antibacterial monomer in combination with UDMA and TEGDVBE to formulate a coating material. Developing this coating material would have the potential to inhibit root caries, especially in the elderly and patients undergoing periodontal surgeries (crown lengthening) with root exposure. Also, it may play a role in reducing the abrasion in the cervical region. Therefore, this study aimed to develop a novel antibacterial resin-based coating to inhibit root caries and evaluate its effectiveness using the antibacterial monomer DMAHDM. We hypothesized that (1) increasing the DMAHDM mass fraction in the cement would not compromise the mechanical and physical properties of the resin-based coating material and (2) the novel antibacterial and hydrolytically stable coating would have potent antibacterial properties, while maintaining a low cytotoxicity comparable to the commercial control. 

## 2. Materials and Methods

### 2.1. Synthesis of Resin Containing Different Mass Fractions of DMAHDM

The formulations of resin used were 55.8% UDMA (Esstech, Essington, PA, USA) and 44.2% TEG-DVBE (mass %), as described by Wang et al. 2018 [15]. Briefly, TEG-DVBE was produced by gradually adding triethylene glycol in dimethylformamide (DMF) to a stirred mixture of NaH in a temperature range of 0 to 4 °C under an argon ambiance for 30 min. Following two hours of agitation, 4-vinylbenzyl chloride in DMF was gradually added over 30 min. The resulting mixture was then agitated at ambient temperature for 18 h. Subsequently, the reaction mixture was neutralized by adding a saturated NH_4_Cl solution (0.6 g/mL of water). The resultant solution was diluted with distilled water and extracted using ethyl acetate. The solvent was evaporated under vacuum, resulting in the formation of a light-colored oil. 

DMAHDM was fabricated via a modified Menschutkin reaction, as previously described [20]. In a 20 mL scintillation vial, 3 g of ethanol was mixed with 10 mmol of 1-bromohexadecane (BHD, TCI America, Portland, OR, USA) and 10 mmol of 2-(dimethylamino) ethyl methacrylate (DMAEMA, Sigma-Aldrich, MO, USA). For 24 h, the vial was agitated at 70 °C. Following the evaporation of the solvent, DMAHDM was obtained in the form of a transparent, colorless, and viscous liquid. The UV resin was combined with DMAHDM to produce resin composites containing DMAHDM at final concentrations of 0%, 3%, 5%, and 7% (by weight). For mechanical reinforcement, 10% of the mass fraction of silanated barium boroaluminosilicate glass particles (d = 1.2 µm, Dentsply Sirona, Milford, DE, USA) was incorporated into the resin. Photoinitiators were then added: 0.2% camphorquinone (CQ, Millipore Sigma, Burlington, MA, USA) and 0.8% 4-N, N-dimethylaminobenzoate (4EDMAB; Millipore Sigma, Burlington, MA, USA).

In this study, Seal & Protect (Dentsply DeTrey GmbH, Konstanz, Germany) was used as commercial control for comparison it comprises penta-, di- and trimethacrylate resins, nanofiller, initiators, a stabilizer, CAHF, acetone, and triclosan. Five coating groups were evaluated:

1. Seal & Protect (designated as “Commercial Control”);
2. UV + 0% DMAHDM + 10% glass (designated as “Experimental control”);
3. UV + 3% DMAHDM + 10% glass (designated as “UV + 3% DMAHDM”);
4. UV + 5% DMAHDM + 10% glass (designated as “UV + 5% DMAHDM”);
5. UV + 7% DMAHDM + 10% glass (designated as “UV + 7% DMAHDM”).

### 2.2. Mechanical Properties

A mold of stainless steel with precise dimensions of 2 × 2 × 25 mm^3^ was utilized to fabricate the resin bars for mechanical testing. The samples were photo-cured (1200 mW/cm^2^, Labolight, DUO, GC, Tokyo, Japan) for 60 s on each side after covering them with mylar strips on both sides to prevent the formation of an air-inhibited layer. The specimens were then stored at 37 °C for 24 h [15]. A 3-point flexural test was used to determine the flexural strength and elastic modulus (n = 6) using a Universal Testing Machine (MTS, Insight 1, Cary, NC, USA) with a 10 mm span and a crosshead speed of 1 mm/min. 

### 2.3. Degree of Conversion

The degree of conversion (DC) was evaluated using an FT-IR spectrometer (Nicolet 6700, Thermo Fisher Scientific, Waltham, MA, USA) (400−4000 cm^−1^ wavelength and 32 scans at 4 cm^−1^ resolution) using the relative band ratio method. The technique estimates the amount of methacrylate groups remaining after exposure to light by measuring the change in intensity of the peak of the C=C vibration at 1637 cm^−1^, with the absorbance peak of the aromatic group at 1583 cm^−1^ as an internal reference [21,22]. A drop of the resin material (mean sd; n = 3) was placed on the attenuated total reflectance (ATR) crystal, covered with a mylar strip, and was then measured before and after light curing for 40 s (1200 mW/cm^2^, Labolight, DUO, GC, Tokyo, Japan).

### 2.4. Paste Flowability

This test was modified to comply with ISO 6876/2012 [23]. Taking into consideration the viscosity of the experimental material, a mass measurement was used instead of volume. Specifically, 50 mg of the resin material of each group (mean ± sd; n = 3) was deposited onto a glass plate with dimensions of 100 × 100 × 3 mm. Another glass plate was then carefully placed on top of it, followed by a 100 g weight. That yielded a total weight of 170 g. After 10 min at room temperature, both the maximum and minimum diameters were measured using a digital caliper (Mitutoyo, Tokyo, Japan). The test was repeated if the discrepancy between the two diameters exceeded 1 mm [24]. 

### 2.5. Streptococcus mutans (S. mutans) Biofilm Model

#### 2.5.1. Resin Samples for Biofilm Testing

Resin discs, each with an 8 mm diameter and 1 mm thickness, were fabricated (n = 6). The photopolymerization process was applied to each sample for 60 s (1200 mW/cm^2^, Labolight, DUO, GC, Tokyo, Japan) on both sides, followed by a 24 h storage at 37 °C following the method of a previous study [25]. The samples were submerged in distilled water and stirred for one hour to facilitate the removal of any remaining unreacted monomers [26]. The resin discs were sterilized with ethylene oxide (Anprolene AN 74i, Andersen, Haw River, NC, USA) and then underwent a seven-day degassing procedure in accordance with the manufacturer’s guidelines to remove any trapped ethylene oxide [27].

#### 2.5.2. Bacteria Inoculation and Biofilm Formation

Bacterial species were authorized for use by the Institutional Review Board of the University of Maryland, Baltimore (HP-00052180). Given its association with dental caries, *S. mutans* (UA159) was chosen as the bacterial species for the present study. *S. mutans* were incubated in brain heart infusion (BHI) broth (Sigma-Aldrich, St. Louis, MO, USA) at 37 °C and 5% CO_2_ for 16 to 18 h. Using a spectrophotometer (Genesys 10S, Thermo Scientific, Waltham, MA, USA), the inoculum concentration was adjusted to 10^7^ colony-forming units (CFU/mL). This adjustment was made based on the standard curve of optical density at 600 nm (OD600) vs. CFU/mL. Each resin disk was placed in the well of a 24-well plate and then covered with 1.5 mL of BHI culture media containing 2% sucrose. The plate was then incubated for 24 h. Afterward, the composite disks were relocated to 24-well plates and then filled with 1.5 mL of new media containing sucrose. They were then left to incubate for an additional 24 h. 

#### 2.5.3. Biofilm Colony-Forming Unit Counts

The biofilm-attached coating disks (n = 6) were transferred into a 24-well plate filled with 1 mL phosphate-buffered saline (PBS), and the biofilms were collected using a combination of scraping and sonication/vortexing. The bacterial suspensions were serially diluted (10^1^–10^6^) and then cultured on BHI agar plates. The agar plates were placed in an incubator for 48 h at a temperature of 37 °C and with a carbon dioxide concentration of 5%. Following incubation, the number of colonies was counted using a loupe (Reichert Quebec Darkfield Colony Counter, Depew, NY, USA), and the counts of biofilm colony-forming units (CFU) were determined and calculated by multiplying the number of colonies by the dilution factor [28]. 

#### 2.5.4. Biofilm Metabolic Activity

The metabolic activity of the biofilm was quantified using a 3-[4,5-dimethylthiazol-2-yl]-2,5-diphenyltetrazolium bromide (MTT) (WST-8, Selleckchem, Huston, TX, USA) test. The resin coating samples, together with the biofilms adhered to them, were moved to a 24-well plate. The plate was filled with 1 mL of MTT dye, which had a concentration of 0.5 mg/mL in PBS. The samples were then placed in an incubator at a temperature of 37 °C, with a 5% concentration of CO_2_, for 1 h. Afterward, every disk was transferred to a fresh 24-well plate with 1 mL of DMSO in each well. The plate was then kept at room temperature (25 °C) in the absence of light for 20 min. To calculate the absorbance, a volume of 200 µL of the DMSO solution was added to each well of a 96-well plate, and the absorbance was measured at a wavelength of 540 nm using a microplate reader (Spectra-Max M5). Higher absorbance readings indicate an elevated level of metabolic activity in the biofilm. The metabolic activity experiment was conducted three times [29].

#### 2.5.5. Lactic Acid Production by Biofilms

The resin composite samples, which had biofilms adhered to them for 48 h, were moved to 24-well plates containing buffered peptone water (BPW, Aldrich, St. Louis, MO, USA). The BPW was supplemented with 0.2% sucrose and the samples were incubated at 37 °C in an atmosphere of 5% CO_2_ for 3 h [30]. Lactate dehydrogenase enzyme was utilized to quantify the lactate levels in the BPW by measuring the optical density at 340 nm using a microplate reader (Spectra-Max M5), as previously explained. The experiment to produce lactic acid was conducted three times [31].

#### 2.5.6. Scanning Electron Microscopy (SEM)

The resin disks with mature *S. mutans* biofilm on their surface were analyzed using a scanning electron microscope (Quanta 200, FEI Company, Hillsboro, OR, USA). The biofilms on the five disks were rinsed with PBS and then stored in 1% glutaraldehyde at 4 °C overnight. Afterward, the samples were rinsed with PBS and dehydrated using a sequence of ethanol treatments. Afterward, they were rinsed with hexamethyldisilazane and allowed to air dry overnight. The samples were sputter-coated with platinum and subsequently examined with a scanning electron microscope.

#### 2.5.7. Human Gingival Fibroblast (HGF) Cytotoxicity

After receiving approval from the University of Maryland, a cytotoxicity assay was conducted using human gingival fibroblasts (HGFs) (hGFBs, CRL-4061, ATCC) due to their proximity to restorative materials. The cells were cultured in fibroblast medium (FM) supplemented with 2% fetal bovine serum, 10,000 units/mL of penicillin, and 10,000 μg/mL of streptomycin. The cells were then inoculated into a 96-well plate at a density of 5000 cells per well, using FM as the medium. The light-cured resin disks were prepared using a mold that is 4 mm in diameter and 1 mm in thickness. The coated disks were then sterilized using ethylene oxide gas and subsequently degassed for seven days. To obtain eluents, each resin disk was submerged in 4 mL of medium for 24 h at a temperature of 37 °C. The ratio of surface area to solution volume was 0.63 cm^2^/mL, falling within the recommended range of 0.5–6 cm^2^/mL set by the International Organization for Standardization (ISO). To assess the cytotoxicity for the anticipated in vivo use, we conducted tests on the original extract solutions as well as a range of dilutions. The original extracts were mixed with fresh medium at dilutions of 1:1, 1:2, 1:4, and 1:8. HGF cells were cultured with 100 µL of the original extracts and their dilutions from each sample for 24 h. The culture media without any extracts were utilized as the negative control for the HGFs. A Cell Counting Kit-8 was used to assess the viability of cells. Following a 24 h incubation period in a 96-well plate, 10 microliters of the CCK-8 solution were applied to each well and the plate was incubated for 2 h at a temperature of 37 °C with a 5% concentration of CO_2_. A SpectraMax M5 was used to measure the absorbance at 450 nm, which indicates the level of live cell dehydrogenase activity in the culture media. Cell viability was quantified as a percentage relative to the control.

### 2.6. Statistical Analysis

The statistical analyses, including normality and power analysis, were performed using Sigma Plot (SYSTAT, Chicago, IL, USA). The data were analyzed using one-way ANOVA and Tukey’s comparison tests to detect significant differences among the groups. The results were considered statistically significant if the *p*-value was <0.05.

## 3. Results

### 3.1. Mechanical Properties

**Figure 1:**
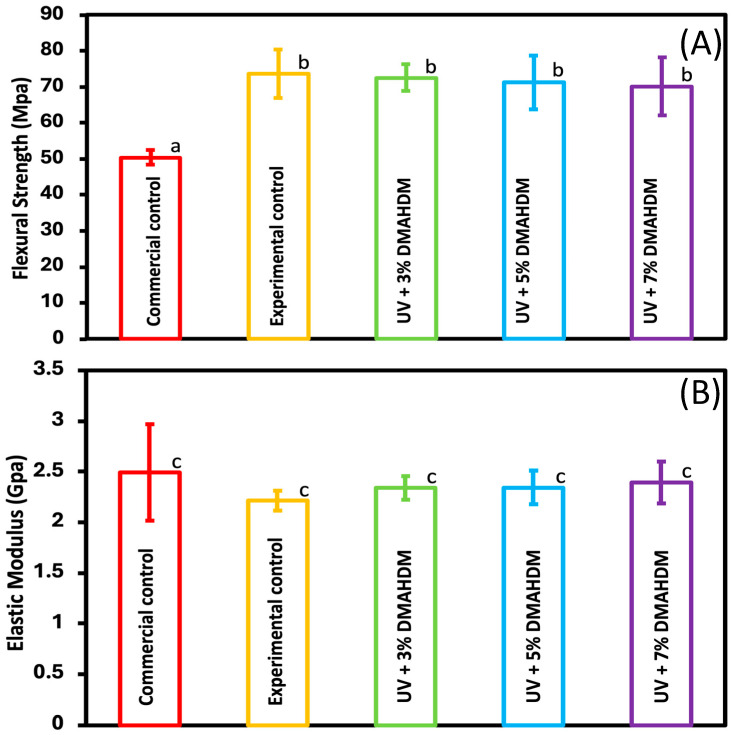
Coating mechanical properties of commercial control, experimental control, and experimental groups: (**A**) flexural strength and (**B**) elastic modulus (mean ± sd; n = 6). Dissimilar letters represent values that are significantly different from one another (*p* < 0.05).

The flexural strength and elastic modulus were quantitatively assessed and are shown in Figure 1 (mean ± sd; n = 6). The incorporation of 0%, 3%, 5%, and 7% DMAHDM with 10% glass fillers resulted in higher flexural strength values compared to the commercial control (*p* < 0.05). The elastic modulus values for all the experimental and commercial groups were not significantly different from each other (*p* > 0.05). The addition of the DMAHDM at any mass fraction did not affect the flexural or the elastic modulus for all the experimental groups.

### 3.2. Paste Flowability

**Figure 2:**
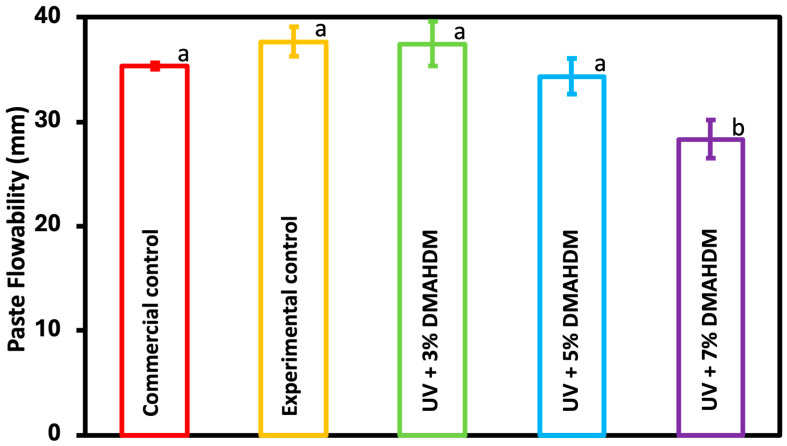
Paste flowability of the commercial control, experimental control, and experimental groups was tested (mean ± sd; n = 3). Dissimilar letters represent values that are significantly different from one another (*p* < 0.05).

The flow properties of the coating resin are presented in Figure 2 (mean ± sd; n = 3). All experimental groups, including the control, exhibited no significant difference when compared to the commercial control (*p* > 0.05), except for the UV + 7% DMAHDM group, which resulted in a notable decrease relative to the other groups (*p* < 0.05). However, it is important to note that the observed decrease in the latter group remained within the range specified by the ISO standard for flowable resin materials.

### 3.3. Degree of Conversion

**Figure 3:**
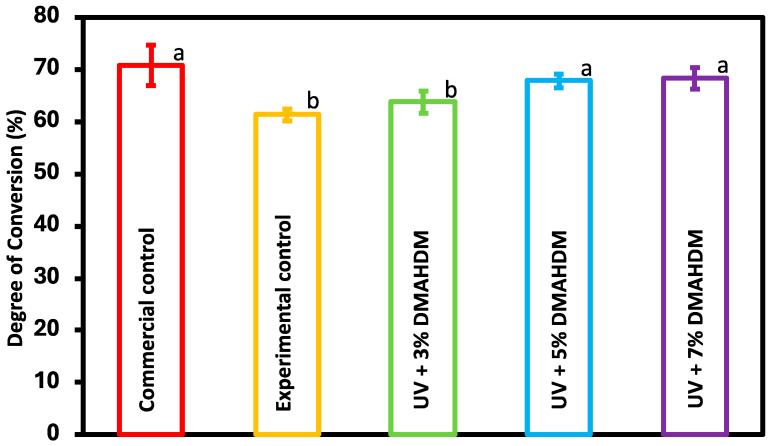
The degree of conversion was assessed for the commercial control, experimental control, and experimental groups (mean ± sd; n = 3). Dissimilar letters represent values that are significantly different from one another (*p* < 0.05).

As shown in Figure 3, the DC of UV + 5% DMAHDM and UV + 7% DMAHDM showed no significant difference compared to the commercial control (*p* > 0.05). However, the experimental control and UV + 3% DMAHDM exhibited a significant reduction when compared to the other groups (*p* < 0.05). However, the lowest DC calculated at 61.8% was within the range deemed clinically acceptable [32].

### 3.4. Biofilm Colony-Forming Unit Counts

**Figure 4:**
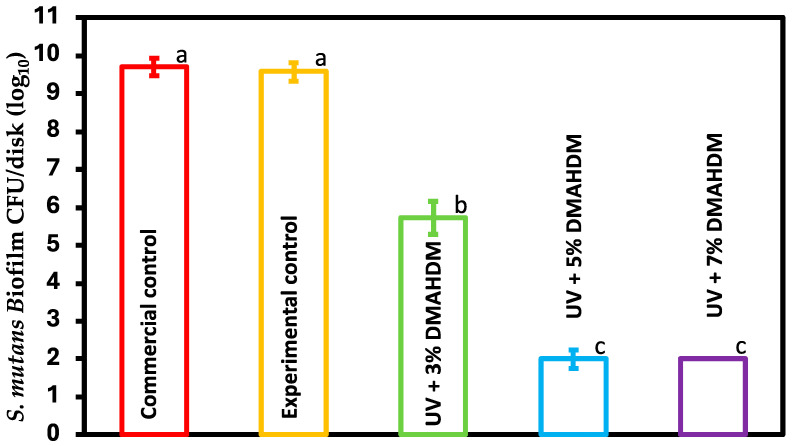
CFU counts of S. mutans biofilms on coating disks of commercial control, experimental control, and experimental groups (mean ± sd; n = 6). Dissimilar letters represent values that are significantly different from one another (*p* < 0.05).

The CFU results using S. mutans biofilms are presented in Figure 4 (mean ± sd; n = 6). The incorporation of 3%, 5%, and 7% DMAHDM resulted in a significant reduction in the CFUs count, with a 4-log reduction for the 3% and 8-log reduction for the 5% and 7% groups. The experimental control showed no statistical difference compared to the commercial control (*p* > 0.05).

### 3.5. MTT Assay of Metabolic Activity of S. mutans Biofilms

**Figure 5:**
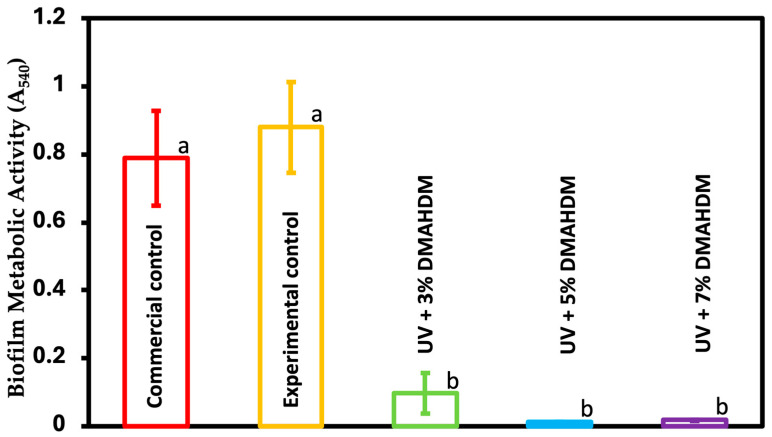
The metabolic activity of the biofilm was assessed using the MTT assay for the commercial control, experimental control, and experimental groups (mean ± sd; n = 6). Dissimilar letters represent values that are significantly different from one another (*p* < 0.05).

Figure 5 depicts the metabolic activity of the S. mutans biofilm formed on the resin disks using the MTT assay after 48 h (mean ± sd; n = 6). The addition of DMAHDM to the resin showed a significant decrease in metabolic activity by approximately 90% compared to the commercial and experimental groups (*p* < 0.05). However, there were no significant differences between the experimental groups (*p* > 0.05)

### 3.6. Lactic Acid Production by S. mutans Biofilms

**Figure 6:**
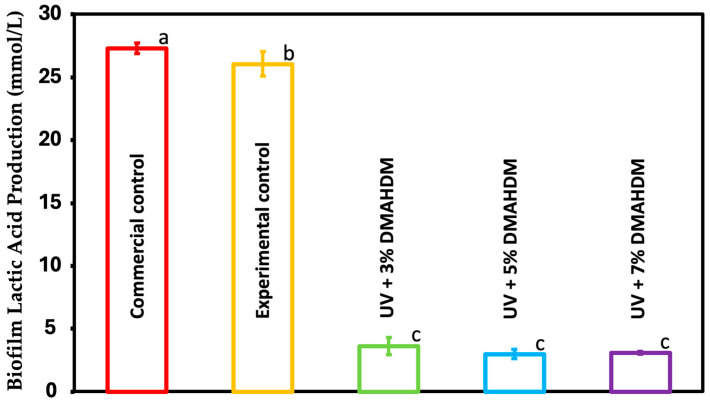
The concentration of lactic acid produced by S. mutans biofilm on coating resin was assessed for the commercial control, experimental control, and experimental groups (mean ± sd; n = 6). Dissimilar letters represent values that are significantly different from one another (*p* < 0.05).

The concentration of lactic acid produced by the S. mutans biofilms is plotted in Figure 6 (mean ± sd; n = 6). The UV coating resin with 3%, 5%, and 7% DMAHDM showed a significant reduction in the lactic acid concentration produced by the bacterial biofilm (*p* < 0.05). The values decreased from 27.2 ± 0.4 mmol/L for the commercial control and 26 ± 0.9 mmol/L for the experimental control to 3.6 ± 0.6 mmol/L for UV + 3% DMAHDM, 2.9 ± 0.3 mmol/L for UV + 5% DMAHDM, and 3 ± 0.1 mmol/L for UV + 7% DMAHDM. There was no significant difference between the commercial and experimental control groups (*p* > 0.05).

### 3.7. SEM Examination of Coating and S. mutans Biofilms

**Figure 7:**
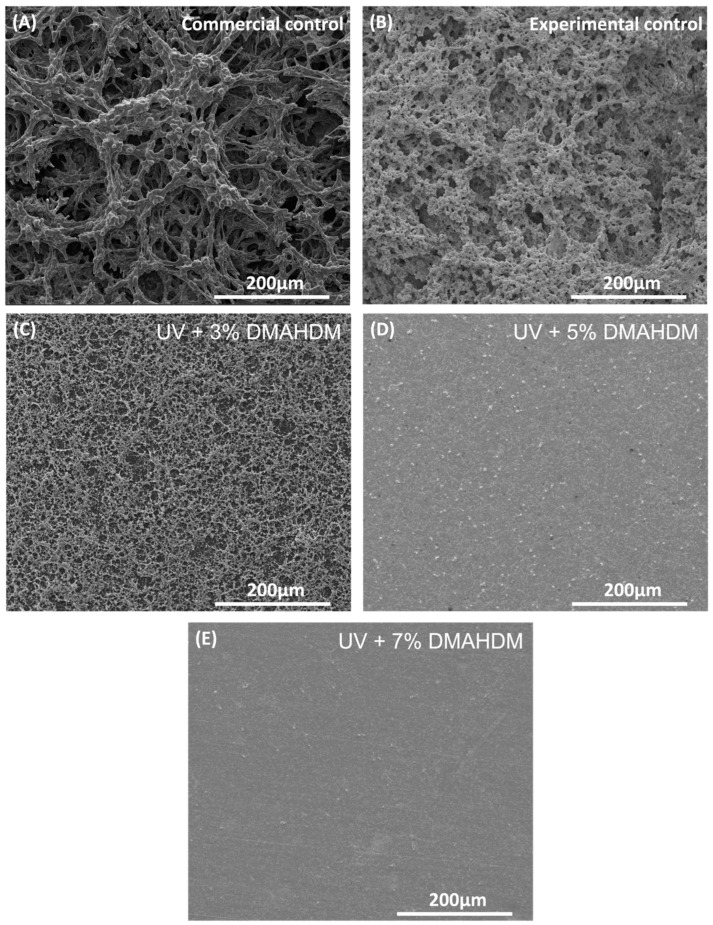
SEM examination of biofilms on resin disks at 48 h. (**A**) Commercial control. (**B**) Experimental control. (**C**) UV + 3% DMAHDM + 10% glass. (**D**) UV + 5% DMAHDM + 10% glass. (**E**) UV + 7% DMAHDM + 10% glass.

Figure 7 shows the SEM results for the S. mutans biofilms formed on the resin disks over two days. Both commercial and experimental control resins were associated with significant biofilm formation, observed as dense, multi-layered microbial communities, and an extensive extracellular polymeric substance (EPS) matrix that covered the surface of the disk. However, including DMAHDM in the UV resin matrix resulted in limited adherence of the S. mutans biofilms, as seen in the resin with 3% DMAHDM where a noticeable reduction in the presence of a biofilm, characterized by a decrease in the number of bacterial colonies and a less dense EPS network compared to the controls. The most significant decrease in bacterial biofilm growth occurred when 5% and 7% DMAHDM were added.

### 3.8. Cytotoxicity

**Figure 8:**
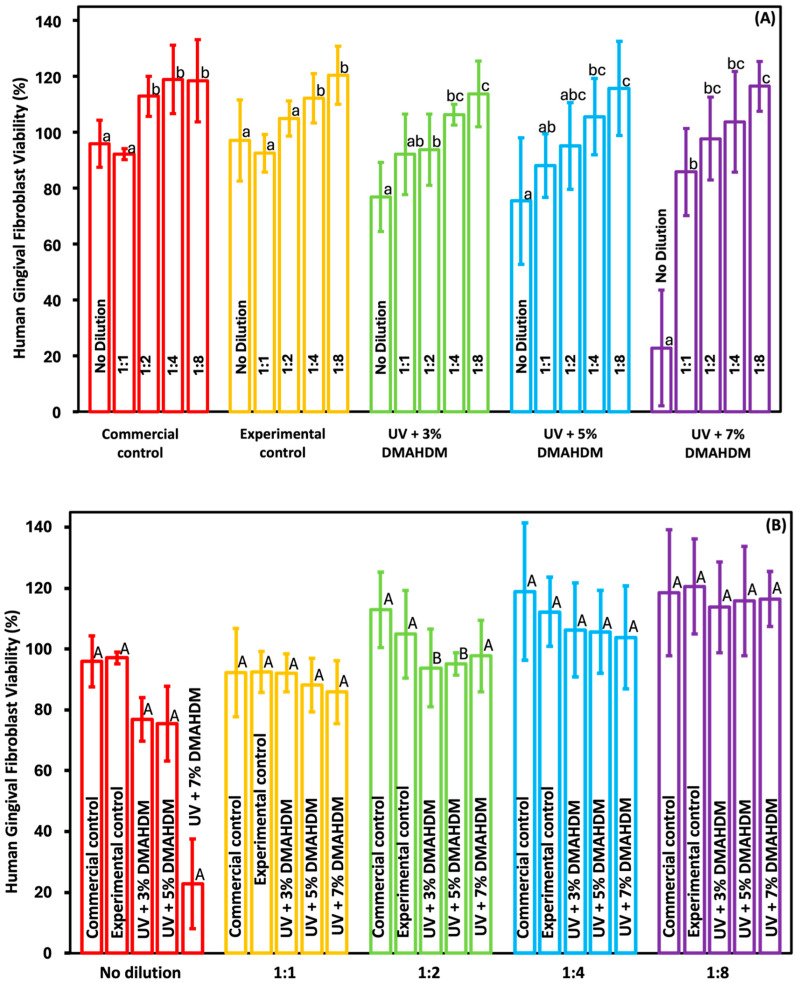
Viability of human gingival fibroblast cells in the presence of the newly developed coating resins (mean ± SD; n = 3 × 3). (**A**) Cell viability for each group; (**B**) viability for each dilution. Dilutions in figures A and B represents the ratio of extract media to fresh fibroblast media. 1:1= 50 μL:50 μL; 1:2 = 33.3 μL:66.6 μL; 1:4 = 25 μL:75 μL; 1:8 = 12.5 μL:87.5 μL. Dissimilar letters represent values that are significantly different from one another (*p* < 0.05).

Figure 8 shows the HGF viability in the presence of the newly developed resin-based coating materials compared to the commercial control (mean ± SD; n = 3 × 3). In Figure 8A, the x-axis represents the various dilutions for each group. Figure 8B displays the comparison between the groups for each dilution. With the exception of the UV + 7% DMAHDM group, none of the groups exhibited any toxicity towards the HGFs. Nevertheless, beginning with the first dilution, all the groups exhibited no statistically significant difference compared to the commercial control (*p* > 0.05).

## 4. Discussion

The present study developed a novel resin-based coating material with potent antibacterial properties through a contact-killing mechanism using DMAHDM at multiple concentrations of 3%, 5%, and 7% without jeopardizing its mechanical and physical properties. This combination could potentially reduce the possibility of developing dental caries, especially on exposed dentin. Furthermore, the presence of filler particles in the material may help in reducing wear and abrasion on the cervical area in elderly patients or those with periodontal diseases [33]. Among the tested concentrations of DMAHDM, the study determined the optimum level of DMAHDM to inhibit the growth of biofilms of *S. mutans*, which is considered one of the most common cariogenic bacteria associated with dental caries [34], without affecting the tested physical and mechanical properties of the UV resin.

All experimental groups with or without DMAHDM showed a significantly higher flexural strength (*p* < 0.05) when compared to the commercial control while maintaining a comparable elastic modulus (*p* > 0.05). The high flexural strength is primarily due to the embedded silanated barium boroaluminosilicate glass particles in the resin formulation. The commercial and experimental groups showed a low modulus of elasticity, which is a desirable factor to consider. Resin-based dental materials with a low modulus of elasticity are believed to exhibit better marginal sealing and prevent microleakages due to the ability of these materials to provide a stress-buffering capacity [35,36,37]. Furthermore, the UV resin used in this study has shown an ability to reduce polymerization shrinkage stress which is a significant issue present in methacrylate-based dental materials [38,39]. We can interpret from these properties that this material would have a better marginal seal and reduce microleakages around the coating.

The flowability of the coating material is an important feature that facilitates its uniform penetration into the tooth structure, establishment of a mechanical barrier to impede microbial growth, and improvement in the material’s durability [40]. The ISO 6876 (2012) specifications were modified to adapt to this experiment. All experimental groups showed excellent flowability that meets the ISO requirement ranging from 26.7 mm to 39.8 mm. The flowability of the UV resin coupled with 3% and 5% DMAHDM, in addition to the 10% glass fillers, did not show any statistically significant difference when compared to the commercial control. On the other hand, a decrease in flowability was observed in the UV + 7% DMAHDM group when compared to the other groups. However, it remained within the limits specified by the ISO specifications. The higher concentration of the antibacterial monomer likely contributed to the increased viscosity observed in the UV + 7% DMAHDM experimental group, which could be due to the effect of the higher percentage of DMAHDM on the filler dispersion within the resin matrix.

The mechanical and physical properties of resin dental materials may be significantly influenced by the polymerization degree of vinyl conversion (DC). A lower DC can result in inferior mechanical and physical properties and lead to the leaching of unreacted monomers which may potentially cause cytotoxicity to adjacent tissues. Typically, the acceptable degree of conversion in dental composites is 55% to 75% [39,41]. The experimental groups UV + 5% DMAHDM and UV + 7% DMAHDM showed a degree of conversion that was comparable to that of the commercial control with no significant differences (*p* > 0.05). However, the experimental control and UV + 3% DMAHDM groups showed a significant difference when compared with the other experimental and the commercial control, groups while still maintaining a DC within the clinically acceptable range (*p* < 0.05). The reason for the difference in the degree of conversion between the experimental groups may be due to the synergistic effects at higher concentrations of DMAHDM which could facilitate a more efficient polymerization process. A higher degree of DMAHDM may have led to a better integration into the polymer network which led to a higher cross-linking density and leaving less unreacted monomers.

Several studies have demonstrated the potent antibacterial properties of DMAHDM monomers when incorporated into composite resins [22,25,42], cement [16], and root canal sealers [43]. Xiao et al. (2019) developed a novel multifunctional nanocomposite for root caries restorations to inhibit periodontitis-related pathogens. They used the same antibacterial monomer (DMAHDM); however, a different resin formulation was developed. Nanoparticles of silver (NAg), 2-methacryloyloxyethyl phosphorylcholine (MPC), and nanoparticles of amorphous calcium phosphate (NACP) were utilized as opposed to our study where DMAHDM was incorporated into UDMA/TEGDVBE resin with 10% glass fillers [42]. The long-term antibacterial effectiveness of DMAHDM incorporated into a low-shrinkage-stress composite resin was evaluated in an in vitro study conducted by Bhadila et al. (2021). The potency of the antibacterial effect was tested against a multi-species biofilm model before and after artificial aging for 90 days. The study found that DMAHDM maintained its antibacterial efficacy after aging [25]. Another article by Wang et al. (2018) investigated the resistance of oral bacteria to multiple antibacterial materials, including DMAHDM. This antibacterial was tested against *Streptococcus mutans*, *Streptococcus sanguinis*, and *Streptococcus gordonii*. The results showed no drug resistance to DMAHDM in any of these bacterial species. The study concluded that DMAHDM did not induce drug resistance and that DMAHDM reduced the number of CFUs by 3–4 logs, with no significant changes over 1 to 20 passages [44]. No paper has investigated the possible effects of this monomer in resin-based coatings. The present study investigated the possibility of adding this monomer to a UV resin to develop a novel coating with antibacterial capabilities to prevent the growth of microbial biofilms; the antibacterial monomer functions through a contact-based bactericidal effect. DMAHDM-containing adhesives with positive charges can inhibit bacterial growth by interfering with bacterial cell membrane activities, disturbing ion exchange, and disrupting the electrical equilibrium of the cell membrane, ultimately leading to the death of bacteria [45]. Multiple antibacterial assays were conducted to test the effect of DMAHDM on the biofilm formed by *S. mutans* on the resin disks. CFU counting was conducted to indicate the viability of the microorganisms. The results showed that adding 5% and 7% DMAHDM to the UV resin resulted in an 8-log reduction compared to the control groups, which was a significant reduction (*p* < 0.05). However, UV + 3% DMAHDM showed a 4-log reduction compared to both controls (*p* < 0.05). The reduction of *S. mutans* biofilm by 3 logs or more is considered of clinical relevance [46].

The MTT assay, which is a colorimetric method used in microbiology to assess cellular metabolic activity as an indicator of cell viability, was performed. A substantial reduction in metabolic activity of around 90% was observed in the resin after the addition of DMAHDM to the UV resin compared to both the experimental and commercial groups (*p* < 0.05). Nevertheless, there were no statistically significant differences among these experimental groups (*p* > 0.05). A lactic acid production assay was also performed to quantify the concentration of lactic acid produced by the bacteria. The UV coating resin containing 3%, 5%, and 7% DMAHDM reduced the concentration of lactic acid produced by the bacterial biofilm by a statistically significant amount (*p* < 0.05). The experimental control had a value of 27.2 ± 0.4 mmol/L, while the commercial control had 26 ± 0.9 mmol/L. UV + 3% DMAHDM reduced the value to 3.6 ± 0.6 mmol/L, UV + 5% DMAHDM to 2.9 ± 0.3 mmol/L, and UV + 7% DMAHDM to 3 ± 0.1 mmol/L. There was no significant difference between commercial and experimental controls (*p* > 0.05). There was a direct correlation between the increase in antibacterial monomer concentration and the reduction in biofilm formation. Moreover, the surface of both the commercial and experimental control samples were shown to exhibit mature growth of *S. mutans* biofilms, as evidenced by the SEM photos. Nevertheless, a notable reduction in the biofilms was seen upon the incorporation of DMAHDM at a concentration of 3%. A clear reduction was visible as the proportion of the antibacterial monomer increased, particularly at a concentration of 7%, where no biofilm formation was detected on the surface. The results of this study are consistent with previous studies when the same monomer was used [17,47]. 

Although this novel material shows highly promising results, it must be non-toxic to human cells to be used in in vivo studies. The cytotoxicity against HGF cells seeded in a 96-well plate with 5000 cells/well was tested. The immersed light-cured resin disks were incubated in the fibroblast media at 37 °C for 24 h with multiple dilutions of the extract media to resemble what would take place intraorally. The typical saliva flow for an average person is 1000–1500 mL/24 h [48], so using only the original extract would not be clinically relevant. All the groups tested were found to be non-cytotoxic according to the ISO recommendations, with a viability higher than 75% [49], except for the 7% antibacterial group, which showed a higher cytotoxicity when undiluted. However, when the first dilution (1:1) was used, the cytotoxicity of the same group immediately decreased, putting it within the acceptable ISO standard. As seen in Figure 8, the toxicity of the material proportionally decreased as the dilution increased. This in vitro study has certain limitations, including the lack of evaluation of fracture toughness (FT), surface roughness (SR), hardness (HC), dentin shear bond strength (DSBS), color stability (CS), and remineralization properties. This bioactive resin material is promising for combating root caries in patients with exposed root surfaces. Further studies are needed to investigate the possibility of adding remineralizing agents to help in the remineralization of lost minerals along with their antibacterial properties. Also, the long-term stability of the coating material on both enamel and dentin needs to be investigated.

## 5. Conclusions

The present study developed a novel bioactive coating material for tooth roots with an 8-log reduction in *S. mutans* biofilm CFUs, and a 90% reduction in bacterial metabolic activity and lactic acid production, without compromising the flexural strength, elastic modulus, paste flowability, and degree of conversion properties of the resin material. This study found that adding 5% DMAHDM to the UV resin is the ideal concentration for achieving a strong antibacterial effect while maintaining the tested physical and mechanical properties compared to other groups. The new coating material had a low level of cytotoxicity against gingival fibroblast cells, matching that of the commercial and experimental controls. This therapeutic coating is promising for the protection of the exposed tooth roots against caries in patients undergoing crown lengthening procedures, periodontal surgeries, or the elderly with gingival recessions.

## Data Availability

The raw data supporting the conclusions of this article will be made available by the authors on request.
